# Short-Term Outcomes and Risk Factors of In-Hospital Mortality in Patients Suffering Acute Mesenteric Ischemia after Cardiac Surgery: Role of Opioids and Lactic Acid

**DOI:** 10.3390/jcm12030857

**Published:** 2023-01-20

**Authors:** Ihor Krasivskyi, Ilija Djordjevic, Mahmoud Tayeh, Kaveh Eghbalzadeh, Borko Ivanov, Soi Avgeridou, Stephen Gerfer, Christopher Gaisendrees, Laura Suhr, Anton Sabashnikov, Christian Jörg Rustenbach, Navid Mader, Fabian Doerr, Thorsten Wahlers

**Affiliations:** 1 Department of Cardiothoracic Surgery, University Hospital Cologne, 50937 Cologne, Germany; 2 Department of Vascular Surgery, Evangelical Hospital Bergisch Gladbach, 51465 Bergisch Gladbach, Germany; 3 Department of Cardiothoracic Surgery, Helios Hospital Siegburg, 53721 Siegburg, Germany; 4 Department of Cardiothoracic Surgery, University Hospital Tuebingen, 72016 Tuebingen, Germany; 5 Department of Thoracic Surgery, University Medicine Essen—Ruhrlandklinik, University Duisburg—Essen, 45239 Essen, Germany

**Keywords:** opioids, critical care, gastrointestinal complications, mesenteric ischemia, cardiac surgery

## Abstract

Acute mesenteric ischemia (AMI) is associated with poor clinical results after cardiac surgery. The aim of this study was to analyse the influence of AMI on short-term outcomes and all relevant risk factors of in-hospital mortality after cardiac surgery. Moreover, we aimed to investigate the role of opioids and lactic acid in the detection and prevention of AMI. Between August 2011 and September 2015, 176 consecutive patients with gastrointestinal complications after undergoing open-heart surgery were identified and included in this study. All patients were divided into two groups: AMI group (n = 39) and non-AMI group (n = 137). In terms of comorbidities, the groups were fairly equal and showed no significant differences. Dialysis was significantly higher (*p* < 0.001) in patients that suffered from AMI. Moreover, gastro-intestinal symptoms such as muscular defense (*p* = 0.004) and the laparotomy rate (*p* < 0.001) were significantly higher in the AMI group. Likewise, in-hospital mortality (*p* < 0.001) was significantly higher in patients with detected AMI. Univariate (*p* < 0.001) and multivariate analysis (*p* = 0.025) of both groups revealed that lactic acid value >2 mmol/L and present treatment with opioids are independent combined predictors of mesenteric ischemia in patients after undergoing cardiac surgery. Moreover, multivariate analysis showed peripheral vascular disease (*p* = 0.004), dialysis (*p* = 0.010), and septic shock (*p* = 0.003) as relevant predictors of in-hospital mortality. Prolonged analgetic treatment with opioids and sudden increase of lactic acid levels are independent combined predictors of mesenteric ischemia in patients after undergoing cardiac surgery. Furthermore, peripheral vascular disease, dialysis, and septic shock are relevant predictors for in-hospital mortality.

## 1. Introduction

Gastrointestinal complications, including acute mesenteric ischemia (AMI), are challenging for health care providers at cardiac surgery units [1]. Moreover, the variety of underlying pathologies which can cause AMI impedes timely treatment [2]. Essential diagnostics and therapeutic measures might be performed delayed as the detection of symptoms is difficult due to the necessary analgesia and sedative treatment [3,4]. Laboratory parameters such as lactic acid are unspecific and do not allow direct implication on the basis of one single diagnostic value [5].

Acute mesenteric ischemia (AMI) is a serious complication with an incidence between 0.1% and 0.5%, and is associated with high mortality rates ranging from 24% to 94% [6]. This feared complication is subdivided into four categories: arterial embolism, arterial thrombosis, mesenteric venous thrombosis, and non-occlusive mesenteric ischemia (NOMI) [6,7]. NOMI is caused by arterial hypoperfusion due to arterial vasoconstriction or low cardiac output syndrome [7]. The bowel injury varies from reversible ischemia with inadequate blood supply to severe inflammatory injury with necrosis and/or perforation [6,8]. Septic shock and death are common complications of AMI [6,9]. In this regard, a timely onset of diagnostics and appropriate therapeutic strategies are crucial for patient’s outcome [10]. The combination of laboratory and clinical parameters might be helpful to detect and prevent fatal AMI [11].

Continuous opioid treatment could affect abdominal malperfusion [12]. It might impair bowel motility and increase the incidence of postoperative paralytic ileus [6,10,12]. Serum lactate might not be elevated in all cases, which creates an additional obstacle to prompt diagnosis and often delays treatment [6,12].

The aim of this study was to analyse the influence of AMI on short-term outcomes and all relevant risk factors of in-hospital mortality after cardiac surgery. Moreover, we aimed to investigate the role of opioids and lactic acid in the detection and prevention of AMI.

## 2. Materials and Methods

A retrospective single center non-randomized study included 156 patients who suffered from gastrointestinal complications. The data was gathered between August 2011 and September 2015. In order to analyse the role of opioids and lactic acid in the prevention and detection of AMI and all relevant risk factors leading to higher mortality, all patients with abdominal complications (n = 176) were divided into 2 groups: AMI group (n = 39) and non-AMI group (n = 137). Our methods were previously described elsewhere [13].

### 2.1. Definition of Gastrointestinal Complications

Gastrointestinal complications were defined as an acute onset of symptoms (meteorism, miserere, diarrhoea, vomiting, muscular defense, and/or silent abdomen) leading to an acute abdomen. Acute abdomen was associated with dysfunction of peristalsis (silent abdomen), circulatory depletion (reduction in extracellular fluid volume that occurs when salt and fluid losses exceed intake on a sustained basis), and hypovolemic and/or septic shock (MAD < 65 mmHg despite catecholamine support). AMI (arterial embolism, arterial thrombosis, mesenteric venous thrombosis, or non-occlusive mesenteric ischemia) was detected and diagnosed with computed tomography angiography [13].

### 2.2. Surgical Approach

Surgical techniques were performed by using the preferences of each surgeon [13]. In order to supply patients with sufficient analgesia after surgery, we used continuous opioid infusion (sufentanil 0.1–1µg/kg) in accordance with the pain scale. In order to provide the sufficient mean arterial pressure (MAD > 65), we used continuous catecholamine infusion (low (0.01–0.1 ug/kg), moderate (0.1–0.3 ug/kg), and high (0.3–0.9 ug/kg) noradrenaline dosage).

### 2.3. Data Collection

The data were analysed after they were withdrawn from our institutional database. All retrospectively analysed variables provided information regarding patients’ demographic, and intraoperative and postoperative data.

### 2.4. Outcomes

The primary endpoint in our study was in-hospital mortality. Secondary outcomes were: acute respiratory distress syndrome (ARDS, PaO_2_/FiO_2_ ≤100 mmHg with PEEP ≥5 cm H_2_O), dialysis, hypovolemic and/or septic shock (MAD < 65 mmHg despite catecholamine support), requirement for mechanical assist device, and duration of mechanical ventilation.

### 2.5. Predictors of Acute Mesenteric Ischemia after Cardiac Surgery

To investigate whether the concomitant circumstance of continuous opioid infusion prior to the diagnosis of AMI and rise of lactate levels is predictive of the occurrence of mesenteric ischemia, lactic acid values on symptom onset were dichotomized into values ≤2.0 mmol/L and >2.0 mmol/L. Continuous opioid infusion (sufentanil 0.1–1µg/kg) and elevated lactate levels (>2.0 mmol/L) were analysed as a combined risk factor for the development of AMI.

### 2.6. Risk Factors of In-Hospital Mortality

To identify all relevant risk factosr of in-hospital mortality, sex, peripheral vascular disease, urgent operation, previous abdominal operation, requiring of mechanical assist device, dialysis, septic shock, duration of mechanical ventilation, high noradrenalin dosage (0.3–0.9 ug/kg), and cardiopulmonary resuscitation were analysed as a combined risk factor of in-hospital mortality.

### 2.7. Ethics

Our study was performed according to the Declaration of Helsinki (as revised in 2013). Ethics Committee of the Medical Faculty of the University of Cologne noted that under the German law we do not need a statement of ethical approval by the local ethics committee in order to conduct retrospective clinical studies.

### 2.8. Statistical Analysis

All data were presented as continuous or categorical variables. Categorical data were expressed as total numbers and percentages. Continuous data were evaluated for normality using one sample Kolmogorov-Smirnov test and were expressed as the mean ± standard deviation (SD) in cases of normally distributed or median (interquartile range) in cases of non-normally distributed continuous variables. Pearson’s χ^2^ or Fisher exact tests were used for comparison of categorical data. Univariate and multivariate analysis were performed using binary logistic regression. *p*-values < 0.05 were considered statistically significant. Statistical analysis was performed using Statistical Package for Social Sciences, version 28.1 (SPSS Inc., Chicago, IL, USA). Our methods have been previously described elsewhere [13].

## 3. Results

**Figure 1:**
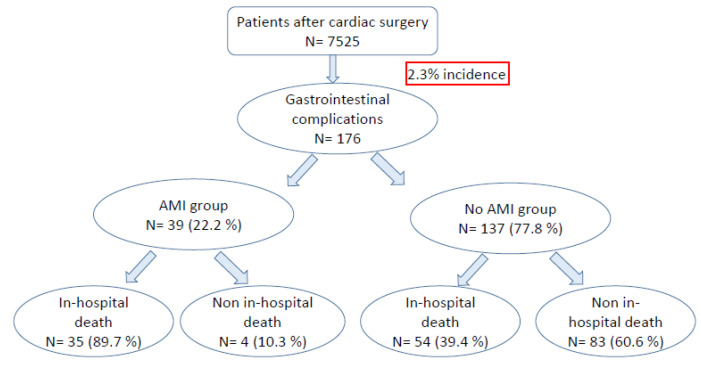
Summarizing plot of patients with gastrointestinal complications after cardiac surgery.

Between August 2011 and September 2015, 7525 patients underwent cardiac surgery (Figure 1). The incidence of gastrointestinal complications accounted to 2.3% (n = 176). AMI was present in 39 patients (22.2%), with an overall-incidence of 0.6% and a pathology-related mortality of 89.7% (n = 35).

### 3.1. Baseline Data

The baseline characteristics of both groups (non-AMI group (n = 137) and AMI group (n = 39)) are summarized in Table 1. There were significantly (*p* = 0.005) more male patients in the non-AMI group (76.6%) than in the AMI group (53.8%). In terms of comorbidities, the groups were fairly equal and showed no significant differences.

### 3.2. Intraoperative Data

Table 2 summarizes the intraoperative data of the two groups (non-AMI group (n = 137) and AMI group (n = 39)). Cardiopulmonary bypass (CPB) time was significantly higher (*p* = 0.007) in the AMI group compared to the non-AMI group. Moreover, cross clamp time was significantly higher (*p* < 0.001) in patients that suffered from AMI. Further intraoperative data did not differ significantly between both groups.

### 3.3. Postoperative Data

The postoperative data of both groups (non-AMI group (n = 137) and AMI group (n = 39)) are summarized in Table 3. Dialysis was significantly higher (*p* < 0.001) in the AMI group compared to the non-AMI group. The gastro-intestinal symptoms such as muscular defense (*p* = 0.004) were significantly higher in patients who suffered from AMI. Moreover, a significantly higher (*p* < 0.001) number of patients from the AMI group underwent laparotomy compared to the non-AMI group. Furthermore, in-hospital mortality was significantly higher (*p* < 0.001) in the AMI group compared to the non-AMI group.

### 3.4. Analysis of Combined Risk Factor: Opioid Infusion and Lactic Acid Elevation

Univariate (*p* < 0.001) and multivariate analysis (*p* = 0.025) of the created patient groups revealed that lactic acid value >2 mmol/L and present treatment with opioids are independent combined predictors of mesenteric ischemia in patients after undergoing cardiac surgery (Table 4).

### 3.5. Combined Risk Factor for In-Hospital Mortality

The combined risk factors for in-hospital mortality are shown in Table 5. Univariate analysis followed by multivariate analysis showed peripheral vascular disease, dialysis, and septic shock as relevant predictors for in-hospital mortality. Sex, urgent operation, previous abdominal operation, and requirement for mechanical assist device had no relevant impact on in-hospital mortality.

## 4. Discussion

The present report describes a single-centre experience with patients suffering from gastrointestinal complications after cardiac surgery. Our analysis, with a specific focus on acute mesenteric ischemia and predictive factors, showed that the combination of lactic acid elevation more than 2 mmol/L and prior opioid treatment with sufentanil (0.1–1 ug/kg) is an independent predictor for AMI development. Furthermore, peripheral vascular disease, dialysis, and septic shock were relevant predictors for in-hospital mortality after open-heart surgery.

As we have already shown, gastrointestinal complications are associated with higher morbidity and mortality after cardiac surgery [13,14,15]. Likewise, in-hospital mortality in our study was 50.5% (n = 89) of the analysed population. Moreover, previous studies stated that abdominal complications strongly impact patient’s outcome after open-heart surgery [16,17]. However, development of the above-mentioned complications is still not well specified due to the multifactorial pathogenesis [18]. In this regard, several factors were found to be associated with adverse gastrointestinal events [19]. The most lethal complication is known to be AMI [13,19]. In this context, the mortality of approximately 89.7% (n = 35) in our cohort is appalling. Current reports corroborate our findings emphasizing bowel ischemia to be associated with extremely poor results and, thus, avoidance of intestinal malperfusion is one of the most important factors to avoid AMI [20,21]. In some clinical situations, the diagnosis of ongoing ischemia is difficult due to necessary analgesia [13,21]. Especially for cardiac surgery patients and prolonged intensive care therapy patients, multimodal analgesia is needed, including opioids [13,20,21].

Several authors mentioned that patients with gastrointestinal complications were older and had more comorbidities compared to patients without gastrointestinal complications [22]. Likewise, the susceptibility of the bowel tract to an ischemic injury due to visceral hypoperfusion in older multimorbid patients could play a crucial role in the development of AMI [13,22,23]. Moreover, surgical trauma and inflammatory response after cardiac surgery could affect abdominal malperfusion [13,24]. In addition, extracorporeal circulation could cause the formation of free radicals, leading to bowel injury [22,25]. Moreover, other authors have stated that prolonged cardiopulmonary bypass time (CPB) might be associated with an increased incidence of AMI after cardiac surgery [13,26]. Likewise, we found significantly higher CPB time (*p* = 0.007) and cross clamp time (*p* < 0.001) in the AMI group compared to the non-AMI group.

Several studies found that dialysis was associated with higher in-hospital mortality after cardiac surgery [1,4,13]. Authors mentioned that kidneys and intestinal function depend on a sufficient perfusion pressure [27]. Therefore, the worsening of renal function could trigger development of abdominal complications [13,27]. Moreover, Djordjevic et al. [13] found that sepsis was an independent predictor of in-hospital mortality after an open-heart surgery. The authors mentioned that, in order to improve vascular dysfunction and capillary leak syndrome, a high dosage of catecholamine was needed [13]. Likewise, dialysis (*p* = 0.010) and sepsis (*p* = 0.003) were associated with significantly higher in-hospital mortality in our study.

Opioid-related adverse events are well known, in particular for the gastrointestinal tract [27]. Medication side effects of administered opioids might impair bowel motility and increase the incidence of postoperative paralytic ileus [28,29]. Opioid-induced constipation has been a focus of several studies in critically ill patients [30,31]. Treatment with methylnaltrexone is controversial, however, as it has been discussed and investigated with incongruent results [32,33].

Reduced bowel function and concomitant inadequate tissue perfusion might lead to elevated lactate levels as a result of anaerobic metabolism [33,34]. Therefore, lactic acid is an important serological parameter for therapy-related decisions regarding diagnostics and therapy in circumstances of gastrointestinal complications in patients after cardiac surgery [35,36].

## 5. Conclusions

Our analysis showed that lactic acid value >2 mmol/L and present treatment with opioids (sufentanil 0.1–1 µg/kg) are combined predictors of mesenteric ischemia in patients after performed cardiac surgery. Furthermore, we found that peripheral vascular disease, dialysis, and septic shock were relevant combined predictors for in-hospital mortality after cardiac surgery. In the diagnostics and treatment of mesenteric ischemia, the combination of these predictive factors should be taken into account.

## 6. Study Limitations

This study has several limitations. Firstly, it was a retrospective single-center analysis, which could lead to lower statistical power. Secondly, we focused on short-term outcomes without paying attention to long-term results and quality of life measures. Thirdly, the data were gathered based on electronic or written patient notes and flowcharts, and were limited to the available variables.

## Figures and Tables

**Table 1:** Patient’s baseline preoperative demographics.

	Non-AMI Group (n = 137)	AMI Group (n = 39)	*p*-Value
Age (years), mean ± SD	71 ± 11	73 ± 11	0.737
Male gender, n (%)	105 (76.6%)	21 (53.8%)	0.005
Euroscore II, mean ± SD	7.7 ± 3.7	8.0 ± 4.1	0.631
Renal insufficiency, n (%)	60 (43.8%)	22 (56.4%)	0.164
PVD, n (%)	23 (16.8%)	10 (25.6%)	0.211
Atrial fibrillation, n (%)	66 (48.2%)	13 (33.3%)	0.123
Previous abdominal surgery, n (%)	35 (25.5%)	12 (30.8%)	0.515
Inotropes before surgery, n (%)	15 (10.9%)	5 (12.8%)	0.776

PVD, peripheral vascular disease.

**Table 2:** Intraoperative data.

	Non-AMI Group (n = 137)	AMI Group (n = 39)	*p*-Value
CABG, n (%)	76 (55.5%)	18 (46.2%)	0.303
Valve surgery, n (%)	53 (38.7%)	18 (46.2%)	0.402
Combined procedure, n (%)	8 (5.8%)	3 (7.6%)	0.462
Urgent procedure, n (%)	27 (19.7%)	9 (23.1%)	0.645
CPB, n (%)	114 (83.2%)	33 (84.6%)	0.835
CPB time (min), mean ± SD	122 ± 59	157 ± 76	0.007
Cross clamp time (min), mean ± SD	66 ± 37	96 ± 51	<0.001
Requirement for MAD, n (%)	37 (27.0%)	6 (15.4%)	0.136

CABG, coronary artery bypass grafting; CPB, cardiopulmonary bypass; MAD, mechanical assist device.

**Table 3:** Postoperative data.

	Non-AMI Group (n = 137)	AMI Group (n = 39)	*p*-Value
Absolut arrhythmia, n (%)	76 (55.5%)	24 (61.5%)	0.500
Dialysis, n (%)	56 (40.9%)	29 (74.4%)	<0.001
CPR, n (%)	7 (5.1%)	5 (12.8%)	0.141
ARDS, n (%)	46 (33.6%)	19 (48.7%)	0.084
Duration of MV, days, mean ± SD	7 ± 12	8 ± 11	0.587
Laparotomy, n (%)	24 (17.5%)	26 (66.7%)	<0.001
Paralytic ileus, n (%)	57 (41.6%)	10 (25.6%)	0.070
Septic shock, n (%)	40 (29.2%)	15 (38.5%)	0.271
Onset of symptoms, PD, median ± SD	6 ± 18	7 ± 31	0.167
Silent abdomen, n (%)	28 (20.4%)	12 (30.8%)	0.174
Abdominal muscular defense, n (%)	3 (2.2%)	6 (15.4%)	0.004
In-hospital stay, days, median ± SD	17 ± 41	16 ± 41	0.616
In-hospital mortality, n (%)	54 (39.4%)	35 (89.7%)	<0.001

ARDS, Acute respiratory distress syndrome; MV, mechanical ventilation; PD, postoperative days; CPR, cardiopulmonary resuscitation.

**Table 4:** Univariate and multivariate analysis of the combined risk factor for mesenteric ischemia: lactic acid elevation and opioid infusion.

Combined Risk Factor	Total Number of Patients	Mesenteric Ischemia	Univariate Analysis *p*-Value	Multivariate Analysis *p*-Value	OR (CI 95%)
Lactic acid <2 mmol/L −opioids	43	4.7% (n = 2)	0.001	0.160	0.280 (0.048–1.651)
Lactic acid <2 mmol/L +opioids	27	14.8% (n = 4)	0.318	0.323	0.566 (0.183–1.749)
Lactic acid >2 mmol/L −opioids	57	22.8% (n = 13)	0.886	0.398	1.699 (0.497–5.806)
Lactic acid >2 mmol/L +opioids	49	40.8% (n = 20)	<0.001	0.025	3.966 (1.188–13.231)

OR, odds ratio; CI, confidence interval.

**Table 5:** Univariate and multivariate logistic regression models of in-hospital mortality.

Combined Risk Factors	Univariate Logistic Regression Model		Multivariate Logistic Regression Model	
	OR (CI 95%)	*p*-Value	OR (CI 95%)	*p*-Value
Sex	1.073 (0.876–3.313)	0.117	1.814 (0.823–3.998)	0.140
PVD	0.373 (0.165–0.839)	0.017	0.241 (0.091–0.637)	0.004
Urgent OP	0.675 (0.322–1.415)	0.298	0.658 (0.275–1.573)	0.346
Previous abdominal OP	1.093 (0.560–2132)	0.794	1.378 (0.597–3.179)	0.452
Requirement for MAD	0.517 (0.255–1.448)	0.067	0.811 (0.897–0.367)	0.811
Dialysis	0.295 (0.159–0.547)	0.001	0.369 (0.173–0.788)	0.010
Septic shock	0.255 (0.127–0.512)	0.001	0.297 (0.134–0.657)	0.003
Duration of MV	1.030 (0.999–1.061)	0.057	1.015 (0.986–1045)	0.305
Noradrenaline (HD)	1.885 (1.411–2.519)	0.001	1.341 (0.939–1.915)	0.107
CPR	0.488 (0.141–1.684)	0.256	0.263 (0.064–1.078)	0.064

MV, mechanical ventilation; AD, assist device; PVD, peripheral vascular disease; MAD, mechanical assist device; HD, high dosage; CPR, cardiopulmonary resuscitation; OP, operation; OR, odds ratio; CI, confidence interval.

## Data Availability

Data can be obtained by a special request.
